# Comparison of Positivity Rates of Rapid Antigen Testing and Real-Time Polymerase Chain Reaction for COVID-19 During the First and Second Waves of the Pandemic in Eastern Uttar Pradesh, India

**DOI:** 10.7759/cureus.16206

**Published:** 2021-07-06

**Authors:** Vivek Hada, Rama S Rath, Aroop Mohanty, Rishabh Sahai, Kanishka Kumar, Subodh Kumar, Hari S Joshi, Surekha Kishore

**Affiliations:** 1 Microbiology, All India Institute of Medical Sciences, Gorakhpur, Gorakhpur, IND; 2 Community Medicine and Family Medicine, All India Institute of Medical Sciences, Gorakhpur, Gorakhpur, IND; 3 Pathology, All India Institute of Medical Sciences, Gorakhpur, Gorakhpur, IND; 4 Internal Medicine, All India Institute of Medical Sciences, Gorakhpur, Gorakhpur, IND; 5 Pulmonary Medicine, All India Institute of Medical Sciences, Gorakhpur, Gorakhpur, IND; 6 Community Medicine and Family Medicine, All India Institute of Medical sciences, Gorakhpur, Gorakhpur, IND

**Keywords:** positivity rate, real-time polymerase chain reaction (rt-pcr), rat, covid-19, second wave

## Abstract

Background

The advent of the second wave of coronavirus disease 2019 (COVID-19) in India caused a new range of challenges in diagnosing the virus. Various point-of-care tests have been introduced for rapid diagnosis. Although rapid antigen tests are the most commonly used, the false-negative rates are high. Therefore, the purpose of this study was to compare the positivity rate of real-time polymerase chain reaction (RT-PCR) testing in rapid antigen-negative cases of COVID-19 during the first and second waves of the COVID-19 pandemic.

Methodology

This was an observational study conducted in the Department of Microbiology, All India Institute of Medical Sciences, Gorakhpur.

Results

In total, 2,168 patients were tested. The percentage positivity rate of the RT-PCR tests among the antigen-negative samples was 4.34% in the first wave of the pandemic whereas it was 8.08% in the second wave.

Conclusions

The main conclusion of this study was that antigen tests should never be used alone for the diagnosis of COVID-19. Instead, they should be confirmed with a RT-PCR test.

## Introduction

The twenty-first century has witnessed three pandemics, all of which have been associated with novel coronaviruses: severe acute respiratory syndrome, Middle East respiratory syndrome, and coronavirus disease 2019 (COVID-19). The recent COVID-19 is caused by a novel zoonotic coronavirus: severe acute respiratory syndrome virus 2 (SARS-CoV-2) [1]. COVID-19 first appeared in Wuhan, one of the largest cities in China, in December 2019 [2]. Considering its alarming rate of spread outside China and a threefold increase in the number of cases, the World Health Organization on March 11, 2020, labeled it a global pandemic [3]. As of May 29, 2021, a total of 168,599,045 confirmed cases of COVID-19 with 3,507,477 confirmed deaths have been reported worldwide [4]. By October 2020, since the first case in December 2019, approximately one million deaths caused by COVID-19 have been documented [5]. This is considered to be the period when the first wave of the highly infectious virus occurred, and unprecedented measures were taken to slow its spread. In India, several molecular laboratories were set up, and existing laboratories were revamped to increase the testing capacity and detect COVID-19 cases [6]. As a result, 1,525 real-time polymerase chain reaction (RT-PCR) laboratories began testing for COVID-19 in India [7]. In addition to testing, self-isolation and proper contact tracing proved highly beneficial in reducing the transmission of COVID-19 [8]. The results of the implemented measures were observable by the end of 2020 when the number of active cases substantially reduced. However, with the advent of the second wave of the COVID-19 pandemic in the early months of 2021, the situation worsened again, bringing a new set of challenges in diagnosing the virus. Because of the sudden increase in the number of cases in both rural and urban settings, various point-of-care rapid antigen tests (RATs) were employed. RATs provide results within 15-20 minutes, enabling COVID-19-positive cases to be identified early, and informing those inflicted to enter isolation as soon as possible [9]. According to the current research, the specificity of RATs is approximately 99.5%, with a false-positive rate of less than 1%, while the sensitivity varies from 0% to 94% [10,11]. Although RT-PCR testing is the highest standard for the detection of COVID-19, it has several limitations. It requires specialized laboratories with molecular virology facilities with specific biosafety and biosecurity precautions, a skilled laboratory staff, specialized equipment, and expensive PCR reagents. In addition, it has time constraints [12]. In this study, we attempted to compare the positivity rates in antigen-negative samples between the first and second waves of the COVID-19 pandemic.

## Materials and methods

This was a secondary data analysis conducted in the Department of Microbiology, All India Institute of Medical Sciences, Gorakhpur. Data were collected from the medical records of outpatients visiting the Microbiology Laboratory for COVID-19 testing. All COVID-19-negative cases identified by an RAT that were received between August 15, 2020 and April 30, 2021 were included in this study. The cases received between August 15, 2020 and February 28, 2021 were included in the first wave of the pandemic, while those received between March 1 and April 30, 2021 were included in the second wave of the pandemic.

Testing methods

**Figure 1:**
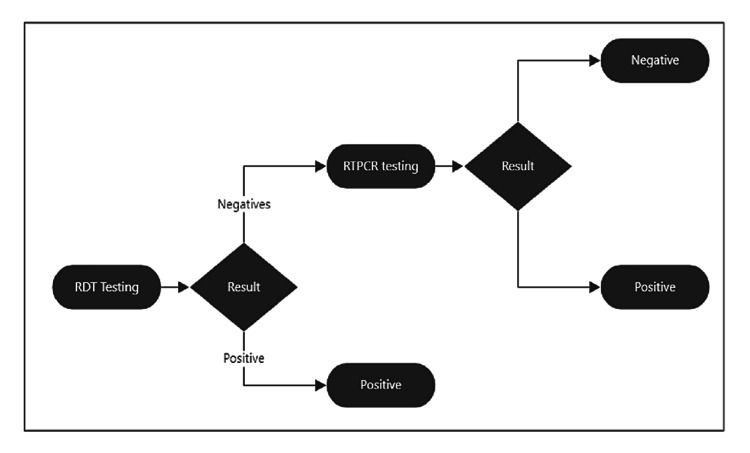
Testing strategy among patients visiting the outpatient department. RTPCR: real-time polymerase chain reaction; RDT: rapid detection test

The commercially available Indian Council of Medical Research (ICMR)-approved rapid antigen detection kits were used for testing, namely, Standard Q COVID-19 (SD Biosensor, South Korea/India), Accucare COVID-19 Antigen Lateral Test Device (MyLab Discovery Solutions, India), Angcard COVID-19 rapid Antigen Test Kit (Angstrom Biotech Pvt. Ltd., India), Alpine COVID-19 Antigen Rapid Test Kit (Alpine Biomedicals Pvt. Ltd., India). In particular, nasopharyngeal swabs were collected and inserted into a pre-filled buffer tube for antigen extraction for 10-15 seconds. The swab was then squeezed and mixed with a buffer. After adequate mixing, one to two drops were applied to the sample wells of the test device. Results were noted after 15-30 minutes. The appearance of both control and test lines denoted a valid positive result, while the appearance of only the control line denoted a valid negative result. The absence of a control line denoted an invalid test, and repeat sampling and testing were performed for invalid test results. Nasopharyngeal and oropharyngeal swab samples were collected in viral transport media for each antigen-negative case and sent for RT-PCR testing. Demographic details such as the age and gender of the patients were retrieved from the records. The testing criteria were the same as those used by the Government of India guidelines. However, in phase 1, both rapid antigen-positive and -negative samples were sent for RT-PCR to validate the findings of the newly established COVID-19 antigen testing laboratory in the institute. The testing guidelines used are shown in Figure 1.

Data compilation

For each patient visiting the testing center, data were collected according to the format prescribed by the ICMR. Further, data related to gender, residence, and test positivity status were extracted using the aforementioned format and were collected from the routine testing database of the laboratory. In this study, all individuals found positive either by an RAT or by an RT-PCR were designated as COVID-19 positive. All individuals found negative by an RT-PCR were classified as COVID-19 negative. The first wave of COVID-19 lasted from August 24, 2020 to February 28, 2021, while the second wave lasted from March 1, 2021 to April 30, 2021.

Data analysis

The collected data were entered into Microsoft Excel 2020 and analyzed using Microsoft Excel and Stata 12 (StataCorp LP, College Station, TX). Descriptive statistics were calculated. Test positivity was represented as a proportion with a 95% confidence interval. The differences in the various parameters between the phases were tested using the chi-square test for categorical variables and a t-test for continuous variables. Statistical significance was set at p < 0.05. As this was a secondary data analysis, no ethical approval was taken. However, permission was obtained from the competent authority. The confidentiality of patient-level data was maintained throughout the study period. All patient-level identifiers were removed during analysis.

## Results

**Figure 2:**
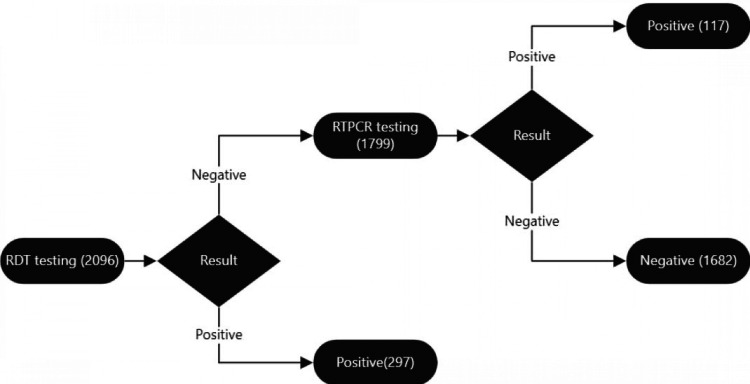
Results of testing at various levels. RTPCR: real-time polymerase chain reaction; RDT: rapid detection test

**Figure 3:**
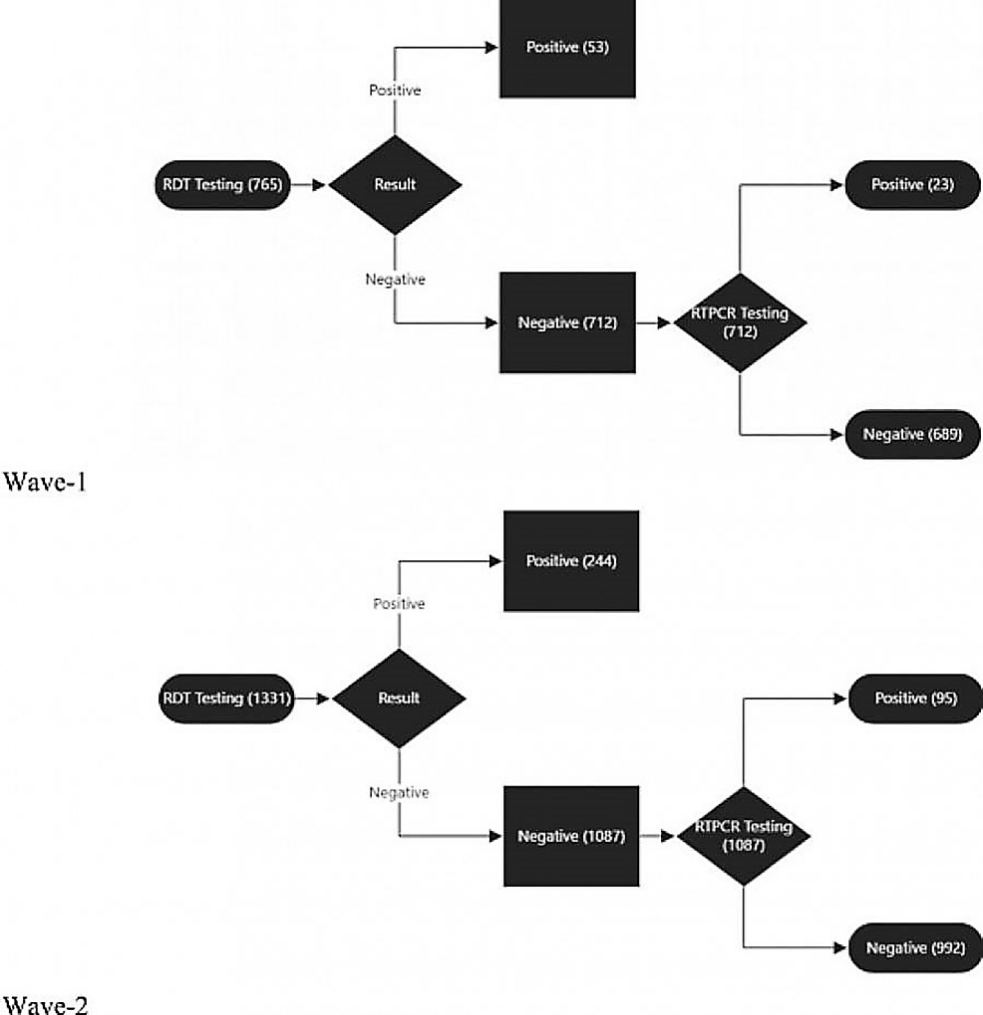
Results obtained in different phases of testing in both the waves. RTPCR: real-time polymerase chain reaction; RDT: rapid detection test

Of the 2,096 patients tested, 297 (14.2%) were found to be positive by RAT and were not tested further according to the ICMR guidelines. The remaining 1,799 (85.8%) cases, which were deemed negative by RATs, were subjected to RT-PCR testing. Among those tested by RT-PCR, 117 (6.5%) were found to be positive and the remaining were negative. Therefore, a total of 414 (20.7%) individuals were found to be positive using these testing methods (Figure 2). In the first wave of COVID-19, 765 RATs were conducted, of which 53 (6.9%) tested positive and the rest were subjected to RT-PCR testing. Of those subjected to RT-PCR testing, 23 (3.0%) were found to be positive. In the second wave of the pandemic, a total of 1,331 RATs were conducted, of which 244 (18.3%) were positive, and the remaining were subjected to RT-PCR. Among those who were subjected to RT-PCR, 95 (7.1%) were found to be positive for COVID-19 (Figure 3).

A comparative analysis of the test positivity rates of RDT and RT-PCR in both waves is summarized in Table 1. The mean age of the participants in the first wave was 31.1 years (SD: 14.0) and in the second wave was 32.6 years (SD: 15.5). The total proportion of female participants was 28.8%, wherein, in the first wave, it was approximately 25.2%, and in the second wave, it was 30.9%. The positivity by RAT was 6.9% (95% confidence interval (CI): 5.3-9.0) and 18.3% (95% CI: 16.3-20.5) in the first and second waves, respectively. This difference is statistically significant. Similarly, the positivity found by RT-PCR among all participants was approximately 3.0% (95% CI: 2.0-4.5) in the first wave and 7.0% (95% CI: 5.8-8.6) in the second wave, which was also statistically significant (p-value: 0.000).

**Table 1:** Differences among patient and test characteristics between the first and second wave of COVID-19. RT-PCR: real-time polymerase chain reaction; RAT: rapid antigen test; COVID-19: coronavirus disease 2019

S. no.	Parameter	Total	First wave	Second wave	P-value
1	Median age (SD)	32.0 (±15.0)	31.1 (±14.0)	32.6 (±15.5)	0.03^*^
2	Female (%)	28.8%	25.2%	30.9%	0.006^#^
3	Total tests done	2,096	765	1,331	
4	Positive tests by RAT	297 (14.17; 12.71-15.73)	53 (6.93; 5.32-8.96)	244 (18.33; 16.34-20.50)	0.000^#^
5	Positive tests by RT-PCR among those who were tested negative by RAT	117 (5.6; 4.7-6.7)	23 (3.0; 2.0-4.5)	95 (7.0; 5.8-8.6)	0.000^#^

## Discussion

This study compared the test positivity results of both waves of COVID-19 in a tertiary care institute in northern India. The results showed that the positivity of the RATs increased from 6.9% to 18.3%, and similarly, the false-negative results increased from 3.0% to 6.0% during the same period. The increase in test positivity in the second wave may be the result of two factors. First, there was an increase in the number of cases, meaning there was an increase in the incidence of the virus in the second wave compared to the first wave. Second, the tests were affected by the changing population characteristics. In the first wave, the population tested included cases with asymptomatic contacts and those who returned to the institute after lockdown. In the second wave, the individuals tested were mainly symptomatic. This might have resulted in a high viral load and higher positivity. Further, there was a higher proportion of females tested in the second wave, which might have resulted in a higher proportion of positivity in the second wave. This may be because of the greater involvement of females in patient care than males [13]. This study observed an increased positivity by RT-PCR in cases that were negative by RAT in the second wave of the pandemic compared to the first wave, emphasizing that RAT should not be used alone. There may be a few possible reasons for this finding. The first is the increase in the caseload in the country and state in the second wave compared to the first wave, which might have resulted in an increased false-positive rate. The second reason for the discrepancy may be due to variations in the virus itself, which may cause a decreased sensitivity and specificity in the rapid antigen kits for disease-causing agents, such as SARS-CoV-2. Wang et al. observed a wide mutation in the target genes used to identify COVID-19 in various regions including India [14]. This might have resulted in the reduced expression of proteins that are targeted by the rapid diagnostic kits. Another point may be the actual test kit used in each wave [15]. In the first wave, the test kits were from the same manufacturer, whereas in the second wave, the kits were from different manufacturers. Although all kits used were approved by the ICMR for the diagnosis of COVID-19, minor differences in sensitivity and specificity might have resulted in the altered negative predictive value of the test kits [16,17]. Overall, a higher viral titer in the affected individuals in the second wave of the pandemic could be one of the causes of the higher positivity rates during the second wave, although only limited data regarding viral load are available.

## Conclusions

This study found an increased positivity by RT-PCR in cases that were found negative by RATs in the second wave of the COVID-19 pandemic compared to the first wave, emphasizing that RATs should not be used alone. False-positive cases were not identified or discussed in this study.

## References

[1] Gupta P, Mohanty A, Narula H (2020). Concise information for the frontline health care workers in the era of COVID-19. Indian J Community Health.

[2] Kumar R, Singh V, Mohanty A, Bahurupi Y, Gupta PK (2021). Corona health-care warriors in India: knowledge, attitude, and practices during COVID-19 outbreak. J Educ Health Promot.

[3] (2021). Director-General's opening remarks at the media briefing on COVID-19 - 19 April 2021. https://www.who.int/director-general/speeches/detail/director-generals-opening-remarks-at-the-media-briefing-on-covid-19-19-april-2021.

[4] (2021). WHO Coronavirus (COVID-19) Dashboard. https://covid19.who.int/?topicsurvey=).

[5] Ioannidis JPA (2020). Global perspective of COVID-19 epidemiology for a full-cycle pandemic. Eur J Clin Invest.

[6] Mohanty A, Kabi A, Mohanty AP, Kumar N, Kumar S (2020). Laboratory diagnosis of COVID-19 infection: current issues and challenges: an Indian perspective. J Adv Med Med Res.

[7] (2021). Total operational (initiated independent testing) laboratories reporting to ICMR. https://www.icmr.gov.in/pdf/covid/labs/archive/COVID_Testing_Labs_28052021.pdf.

[8] Kucharski AJ, Klepac P, Conlan AJK (2020). Effectiveness of isolation, testing, contact tracing, and physical distancing on reducing transmission of SARS-CoV-2 in different settings: a mathematical modelling study. Lancet Infect Dis.

[9] Mohanty A, Kabi A, Kumar S, Hada V (2020). Role of rapid antigen test in the diagnosis of COVID-19 in India. J Adv Med Med Res.

[10] Scohy A, Anantharajah A, Bodéus M, Kabamba-Mukadi B, Verroken A, Rodriguez-Villalobos H (2020). Low performance of rapid antigen detection test as frontline testing for COVID-19 diagnosis. J Clin Virol.

[11] Dinnes J, Deeks JJ, Adriano A (2020). Rapid, point-of-care antigen and molecular-based tests for diagnosis of SARS-CoV-2 infection. Cochrane Database Syst Rev.

[12] Mustafa Hellou M, Górska A, Mazzaferri F (2021). Nucleic acid amplification tests on respiratory samples for the diagnosis of coronavirus infections: a systematic review and meta-analysis. Clin Microbiol Infect.

[13] Dwyer JW, Seccombe K (1991). Elder care as family labor: the influence of gender and family position. J Fam Issues.

[14] Wang R, Hozumi Y, Yin C, Wei GW (2020). Mutations on COVID-19 diagnostic targets. Genomics.

[15] Maitra A, Sarkar MC, Raheja H (2020). Mutations in SARS-CoV-2 viral RNA identified in Eastern India: possible implications for the ongoing outbreak in India and impact on viral structure and host susceptibility. J Biosci.

[16] (2021). Ministry of Health and Family Welfare. Indian IVDs Industry through the pandemic. https://dbtindia.gov.in/sites/default/files/uploadfiles/Indian%20IVDs%20Industry%20Through%20the%20Pandemic%20E-Brochure-23rd%20Dec%202020.pdf.

[17] Yamayoshi S, Sakai-Tagawa Y, Koga M (2020). Comparison of rapid antigen tests for COVID-19. Viruses.

